# Chronic Epstein Barr virus infection leading to classical Hodgkin lymphoma

**DOI:** 10.1186/s12878-016-0059-3

**Published:** 2016-07-19

**Authors:** Nawid M. Sarwari, Joseph D. Khoury, Cristhiam M. Rojas Hernandez

**Affiliations:** Department of Internal Medicine, University of Texas, Houston Health Sciences Center, Houston, TX USA; Department of Hematopathology, University of Texas MD Anderson Cancer Center, Houston, TX USA; Section of Benign Hematology, University of Texas MD Anderson Cancer Center, Houston, TX USA

**Keywords:** Epstein Barr Virus, Lymphoma, Lymphadenopathy, Cytopenias

## Abstract

**Background:**

Chronic Epstein Barr virus (EBV) infection in an immunocompetent host has been described however it is not a common entity. It has been linked to many lymphoproliferative disorders and achieves such via many molecular mechanisms, some of which are poorly understood. In addition to infectious mononucleosis, the EBV is linked to various other hematological pathologies and autoimmune disorders.

**Case presentation:**

We describe the case of an elderly immunocompetent female who presented with non-specific symptomatology, lymphadenopathy, cytopenias, elevated autoantibody titers and a crescent EBV viral load that were suggestive of a multisystemic inflammatory disease related to EBV. Extensive work up including multiple bone marrow biopsy and lymphoid tissue procedures ultimately led to the diagnosis of Hodgkin lymphoma.

**Conclusion:**

EBV-related lymphomagenesis is complex and through the utilization of its nuclear antigens and latent membrane proteins the virus is able to shape the microenvironment to promote the various pathologies seen. Moreover, the diagnosis of EBV-associated lymphoproliferative disorders might be challenging when they present in immunocompetent individuals. Our case also represents an emphatic reminder for clinicians that spontaneous regression of lymphadenopathy is not exclusive of low-grade lymphoid malignancies.

## Background

Infectious mononucleosis is considered a common worldwide infection, with a lifetime prevalence of 90 %. EBV is the major cause of infectious mononucleosis 90 % of the time. It is a double stranded DNA virus that belongs to the family Herpesviridae and subfamily Gamma herpesviridae [1]. Clinically many entities may mimic infectious mononucleosis thus careful examination and history taking must ensue to ensure proper diagnosis and management. It is defined as the classical triad of fever, pharyngitis, and cervical lymphadenopathy along with lymphocytosis. The challenge herein lies in the nonspecific prodromal symptoms such as fevers, chills, and malaise which may mimic various other bacterial, viral, or fungal infections. The lymphadenopathy may be prominent in the anterior as well as the posterior triangles of the neck. Furthermore, certain patients may exhibit palatal petechiae, splenomegaly, hepatomegaly, and jaundice.

The acute viral infection generally resolves within 2–4 weeks. Most cases can be treated with conservative measures such as rest, hydration, analgesics, and antipyretics. In most cases of infectious mononucleosis, splenomegaly resolves by 4–6 weeks.

Less commonly, EBV infection may cause further neurological and hematological complications such as immune mediated cytopenias. EBV has been implicated in several autoimmune diseases, most notably systemic lupus erythematosus, rheumatoid arthritis and multiple sclerosis. It is postulated that EBV may be an early environmental trigger of such autoimmune diseases [2].

The virus can remain in a latent status with genome persisting in the nucleus of infected cells in the form of non-integrated circular episomes that have a nucleosomal pattern similar to the host chromatin. Through histone modification and DNA methylation, viral promoters are regulated for the lytic cell cycle reactivation. The EBV latent episomes have also the capacity to influence the epigenetic state of the host DNA and result into modulation of tumor suppressor gene expression and carcinogenesis, including lymphomas [3].

The lymphoproliferative disorders associated with EBV include Burkitt lymphoma, and Hodgkin lymphoma. Other described types of malignant neoplasms associated to EBV include plasmablastic lymphoma, EBV-positive diffuse large B-cell lymphoma, primary effusion lymphoma, classical Hodgkin lymphoma, T/NK lymphoproliferative disorders and lymphomatoid granulomatosis [4, 5].

## Case presentation

A 69 year old Hispanic female with a past medical history significant for hypertension, diabetes mellitus, coronary artery disease status post revascularization, ischemic cardiomyopathy and chronic kidney disease had presented to our hospital for further evaluation and care. For the past few months the patient had suffered weight loss (approximately 30–35 lb), generalized malaise, low grade fevers (99.9 F), non-productive cough, myalgias and arthralgias. She did not endorse any night sweats or recent travel history, and there were no sick contacts at home. Prior to admission to our institution the patient was at an outside hospital and was being treated for sepsis with combinations of antibiotics including intravenous vancomycin, meropenem, echinocandin, metronidazole, and piperacillin tazobactam for 2 to 3 weeks duration as she was developing fevers and productive cough.

The physical exam yielded a thin and chronically ill appearing female patient.

Vital signs at the time of admission were notable for hypertension (186/79 mmHg). Her eyes did not show any conjunctival hemorrhaging or icterus, however there was conjunctival pallor. Oropharynx did not show any active exudates or ulcerative lesions. Her cardiac exam did not reveal any murmurs and her airways were clear to auscultation. Abdominal exam revealed palpable splenomegaly. Examination of the extremities did not show nail deformities including splinter hemorrhages or nail pitting. Her skin had no evidence of skin nodularities or other lesions. There was a 1 cm non tender, movable left axillary lymph node. The rest of the physical examination was unremarkable.

Initial laboratory work revealed that the patient had pancytopenia. White blood cell count (WBC) was stated at 2.4 K/uL, absolute neutrophil count (ANC) 1.92 K/uL, hemoglobin (Hgb) of 11.6, and platelet count of 39,000/uL. B2-microglobulin was seen elevated at 9.2 mg/L. Basic metabolic panel obtained showed sodium 134 mEq/L, Potassium 3.5 mEq/L, Chloride 103 mEq/L, Bicarbonate 26 mEq/L, Blood urea nitrogen 30 mg/dl, Creatinine 0.90 mg/dl, glucose 220 mg/dl. Liver function panel showed Albumin 2.2 g/dl, normal transaminases, total bilirubin 0.8 mg/dl, direct bilirubin 0.4 mg/dl. Urine analysis showed 200 mg/dl of protein and occasional amorphous crystals.

**Fig. 1:**
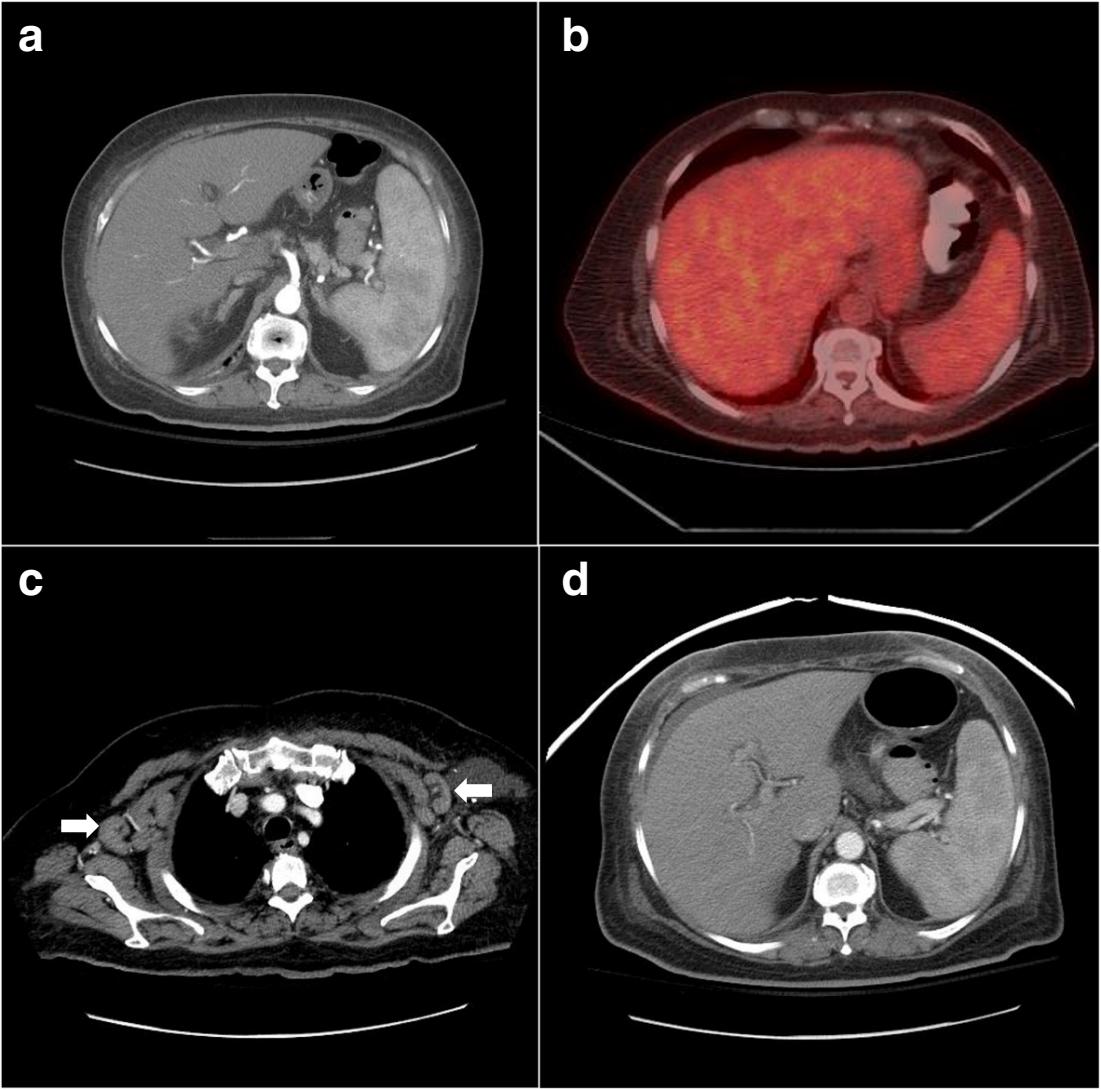
Imaging findings at disease presentation and progression. **a** CT abdomen and pelvis with contrast showing an enlarged spleen with micronodular pattern. **b** PET-CT imaging showing interval resolution of splenomegaly and documenting no evidence of hypermetabolic adenopathy. **c** Repeat CT chest with contrast evidencing development of a right axillary node measuring 2.9 × 2.4 cm and a left axillary node measuring 3 × 1.5 cm (white arrows). **d** Repeat CT abdomen and pelvis evidencing again an enlarged spleen with numerous small subtle hypodense nodules

Initial computed tomography (CT) scan of the abdomen and pelvis during hospital admission showed a 15 cm spleen which was heterogenous and micronodular, as well as scattered abdominal and pelvic lymphadenopathy (Fig. 1a). No imaging signs of portal hypertension were noted.

During the hospitalization our patient was noted to become more confused and agitated, and there was concern that the patient was having seizures as she was seen numerous times to lose consciousness and awaken, with occasional loss of bladder function. A brain magnetic resonance imaging (MRI) showed subcortical non enhancing FLAIR hyper intense foci in the bilateral posterior occipital lobes.

Lumbar puncture and cerebral spinal fluid (CSF) studies were further performed for further analysis, and showed: WBC 1; no red blood cells; protein 62 mg/dL; glucose 35 mg/dL; and, lactate dehydrogenase 234 mIU/mL. Most notable negative findings in the CSF included no detectable viral cultures for cytomegalovirus, adenovirus, herpes virus, and varicella zoster virus, negative cryptococcal antigen serology and negative bacterial and fungal cultures.

Electroencephalogram studies showed triphasic morphology waves with and without sharp negative component at 2–3 hertz. These were seen in continuum mostly with subtle waxing and waning features. To 0.5 mg intravenous alprazolam, above discharges had slowly resolved, replaced by organized background that consists of 6 hertz rhythms. Importantly, there was a clinical improvement after alprazolam; suggesting a nonconvulsive seizures responding to benzodiazepine.

Two weeks later after the initial CT imaging and after the patient was more stable from the neurological perspective in order to attempt a lymph node excisional biopsy, a PET-CT imaging was performed and showed complete resolution of the previously detected lymphadenopathy and splenomegaly. (Fig. 1b).

Additional serologies for viral hepatitis and autoimmune etiology work up were performed. A bone marrow biopsy was performed and additional serum erythropoietin level, iron profile; cobalamin and folate level were determined.

The patient returned to the benign hematology clinic after discharged from the hospital. During her follow up appointment she appeared in better spirits and recovering well. Physical examination was notable for an absence of lymphadenopathy particularly in the cervical and axillary region, as well as no palpable organomegaly in the abdomen. It was further revealed that her antinuclear antibody (ANA) titer was highly positive (1:640), ESR 108 mm/hr with a CRP 6.74 mg/L. Negative viral hepatitis B, C and Human Immunodeficiency virus serologies.

Serum quantitative immunoglobulin showed a mildly elevated Ig A and Ig G level, without other abnormalities. Serum and urine protein electrophoresis did not reveal a monoclonal gammopathy. Serum free light chain levels showed kappa 96.3 mg/L and lambda 60.06 mg/dL with a normal ratio 1.6 in a patient with chronic kidney disease.

Complement C3 level was normal with a slightly elevated C4 at 46 mg/dL.

Bone marrow aspirate yielded a predominance of CD3 positive polytypical small T cells and a population of polyclonal B-cells by flow cytometry and negative for lymphoma or other malignant process. A focal lymphohistiocytic aggregate with granuloma formation was noted; occasional larger cells with prominent nucleolus identified; eosinophils were increased mildly in the vicinity of this aggregate. Acid fast bacilli and fungal stains were attempted; however the focal granuloma could not be appreciated at subsequent sections.

The patient’s pancytopenia was thought to be likely secondary to systemic lupus erythematosus and she was thus referred to rheumatology for further work up, and further testing included anti-double-stranded DNA, anti-RNP, anti-CCP antibody, ANCA vasculitis panel, direct antiglobulin test, lupus anticoagulant, anticardiolipin antibody, anti-double-stranded DNA antibody, and anti-SSA and anti-SSB. All these tests were negative, thus ruling out systemic erythematous lupus, rheumatoid arthritis, and other connective tissue disorders.

The interval improvement and self-limited clinical course in our case led us to believe that her elevated ANA titers, her bone marrow granuloma were secondary to an EBV infection-related syndrome*.* Monospot test was not performed and at that point we evaluated for EBV PCR and EBER staining in the bone marrow. Initial EBV PCR results were positive (960 copies/mL), while Epstein–Barr virus-encoded small RNAs staining in the bone marrow was negative. Our in situ hybridization stain is validated to work on decalcified bone marrow trephine biopsies and all stains include an on-slide positive control. As such, the likelihood of a false negative result is low. A possible explanation includes a sampling factor with lack of infected B-cells in the bone marrow despite the presence of viremia.

Her follow up labs during subsequent clinic visits showed WBC 5.3 K/uL, ANC 3.15 K/uL, Hgb 7.4 g/dL, calculated reticulocyte index of 1.1 and platelets 172 K/uL. Iron was 68 ug/dL, transferrin 124.1 mg/dL, ferritin 2952 ng/mL and serum erythropoietin levels were 31.7 mIU/mL. Serum cobalamin was 394 pg/mL and serum folate 12.3 ng/mL. Since there was spontaneous interval resolution of her neutropenia and her thrombocytopenia our diagnosis at that point was cytopenias secondary to infectious mononucleosis and anemia of chronic kidney disease related to diabetes.

**Fig. 2:**
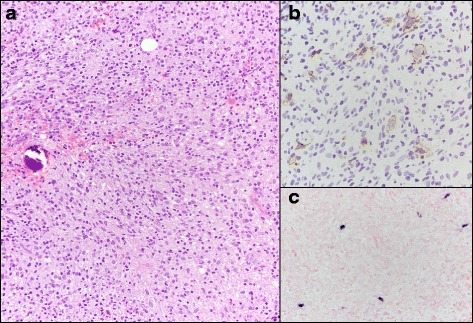
Bone marrow histopathologic findings confirming diagnosis of Hodgkin lymphoma. **a** Bone marrow biopsy demonstrated involvement by classical Hodgkin lymphoma. Neoplastic large lymphoid cells with Hodgkin-Reed-Sternberg morphology were present in a background rich in histiocytes, small lymphocytes, and plasma cells. **b** The neoplastic cells were positive for CD30. The neoplastic cells were also positive for the B-cell marker PAX5 by immunohistochemistry (not shown). **c** Colorimetric in situ hybridization was positive for Epstein-Barr virus-encoded RNA within the neoplastic cells (blue signal)

Our patient presented 7 weeks later at the emergency room with a recurrent febrile syndrome with diffuse cervical and axillary lymphadenopathy and splenomegaly (Fig. 1c-d) and worsening cytopenias: WBC 1800/uL, ANC 1480/uL, Hgb 8.3 gr/dL, platelet count 20,000/uL; a repeat EBV titer revealed 6580 copies/mL. CT imaging with contrast found bilateral axillary, mediastinal and hilar lymphadenopathy. Low volume retroperitoneal and pelvic lymphadenopathy, hepatosplenomegaly with numerous small ill-defined hypodense lesions was in the spleen and probably also within the liver (Fig. 1d). At this time a repeat bone marrow biopsy and an excisional lymph node biopsy were performed and reviewed. The bone marrow specimen showed a solitary large cell positive for CD30 identified in the clot. No atypical cells are identified in core biopsy as assessed by IHC for CD30 and Pax-5. EBER was positive in few and scattered cells. The lymph node sections demonstrated near-total effacement of the lymph node architecture by a neoplasm comprised of neoplastic cells with Hodgkin-Reed-Sternberg morphology. The neoplastic cells were positive for CD15, CD30, PAX5 (weak), and MUM1. They were negative for CD3, CD20, CD45, ALK, and EMA. The findings were diagnostic of classical Hodgkin lymphoma, best subtyped as lymphocyte-depleted. (Fig. 2a-c). The expression of PAX5 with weak intensity coupled with MUM1 expression by the neoplastic cells is diagnostic of classical Hodgkin lymphoma. The expression of PAX5, a gene encoding a B-cell-specific transcription factor, excluded T-cell lymphoma as a diagnostic consideration. It is worth noting also that flow cytometry done on a bone marrow sample with involvement by lymphoma showed no evidence of T-cell or B-cell immunophenotypic aberrancies.

The patient was started on ABVD chemotherapy regime, bleomycin was held secondary to patient’s history of cardiomyopathy. She received three cycles of chemotherapy with complete clinical remission. Additional cycles of chemotherapy were not completed given prolonged and severe chemotherapy-induced cytopenias that eventually recovered over the following months. A bone marrow biopsy at completion of treatment revealed no morphologic evidence of residual Hodgkin lymphoma. Her most recent surveillance PET-CT imaging, nine months from completion of treatment, showed no hypermetabolic lymphoma and continuing complete metabolic response to therapy.

## Discussion

Hereby we have presented a challenging case of EBV infection leading to Hodgkin lymphoma, which initially manifested as a syndrome consisting of fever of unknown origin, lymphadenopathy, elevated ANA titer and cytopenias with spontaneous interval resolution suggesting infectious mononucleosis versus an autoimmune disorder. In an adult patient complaining of chronic fever with no true identifiable source, autoimmune disorders and etiologies including infectious endocarditis, intra-abdominal abscess and viral pathogens such as HIV are within the differential diagnoses. Occult malignancies are also etiologies of such a clinical presentation, especially in elderly population [6–8].

Roughly one-third of healthy patients have a positive ANA titer of 1:40, and healthy woman are said to have a more likelihood of positive titers relative to men. Other non-rheumatological diseases such as thyroiditis, hepatitis, malignancies, infections, environmental exposures, and prescription drugs may show the presence of ANA [9]. Acute EBV infection or reactivation is correlated with the presence of high ANA antibodies which gives way to the notion that the virus causes the dysregulation of the immune response towards self-antigens. There is a certain level of molecular mimicry in inducing auto-antibodies and the immune response towards EBV [9].

The next diagnostic challenge in our case was the interpretation of the presence of granulomata in the bone marrow. A variety of disorders have been implicated in the pathogenesis of bone marrow granuloma. They include malignant lesions; viral, bacterial, and fungal infections; autoimmune diseases; drugs; and sarcoidosis [10]. The presence of non-caseating granulomata has been described and associated with Hodgkin lymphoma and non-hodgkin lymphoma [11–14]. In Hodgkin lymphoma, the frequency of involvement by granulomata in the bone marrow is not clearly determined, and some series have described it in up to 10 % of cases [14]. It seems that the presence of granulomata does not translate into a major difference on survival outcomes [12].

The EBV is associated with lymphoproliferative disorders (PTLD) that comprise a vast array of lymphoid proliferations after the organ transplant period. The patients affected comprise mainly those who have received either solid organ or bone marrow transplantation. The clinical spectrum ranges from that which mimics an inflammatory reaction to more aggressive and lethal B cell proliferations that often resemble non-Hodgkin lymphoma. The pathogenesis described involves the donor derived B cells which have been previously infected by EBV. With the organ recipient having insufficient T cell function, there is uninhibited growth of EBV infected cells. Multiple genetic mutations ensue which ultimately leads to virus-independent cell growth, which often exhibit more aggressive forms of disease. These tumors typically involve the transplanted organ or other regions of the gastrointestinal tract, and the incidence shows a direct relationship to the severity and duration of the immunosuppressive therapy [4]. Our case is unique in several aspects. The patient did not have a history of solid organ or hematologic transplant and she was not on pharmacologic immunosuppression. Her only risk factor for immune compromise was her history of diabetes. Her syndrome initially resembled the more benign subtypes of the PTLD which are characterized as more inflammatory and reactive with the regression and spontaneous resolution of lymphadenopathy and splenomegaly, however with time the aggressive malignancy was seen on biopsy as a rare entity: lymphocyte depleted Hodgkin lymphoma (HL).

Of the various subtypes of HL, the lymphocyte depleted classical Hodgkin Lymphoma (LDCHL) type is the rarest and so consequentially little information is known about its clinical characteristics, course, and treatment outcomes. Less than 1 % of all patients with HL are diagnosed with LDHL. The diagnoses requires immunohistochemical staining which can show one of two subtypes of LDCHL; Reed-Sternberg (RS) cells in a hypocellular background with disordered fibrosis, and rich in histiocytes, or the reticular variant showing numerous RS cells with bizarre cytologic features [5].

Unfortunately patients with the LDCHL tumor often have more unfavorable characteristics such as more advanced stage, B symptoms, mediastinal mass, extranodal disease, high ESR, and more lymph node involvement. Furthermore patients tend to have more bone marrow and liver involvement. Patients also have lower rates of complete remission, progression free survival (PFS), and overall survival (OS) relative to other patients with other HL subtypes. Clinically those with the LD subtype have poorer prognostic factors and poor overall outcomes [5]. Our case demonstrates the broad spectrum of EBV-related lymphoproliferative neoplasms and the occurrence in a patient without the classic immunosuppression risk factors.

Finally, the prevalence and prognostic significance of EBV infection in classical Hodgkin lymphoma has been described in a recently published meta-analysis. It found that the prevalence of EBV is more common in males and typically seen in pediatric population, predominantly found in the mixed cellularity subtype and in patients with advanced clinical stage. However, the presence of EBV had little effect on patient’s overall and event-free survival [15].

## Conclusion

Our case illustrates one of the different clinical features in the spectrum of EBV infection. Age-related EBV-associated lymphoproliferative disorders represent a well described complication of EBV infection, typically seen in immunosuppressed individuals [16]. Our case describes an EBV-related syndrome in an immunocompetent host that mimicked features of a transient viral illness and a systemic inflammatory disorder. Ultimately, it declared itself with a pattern of chronicity that led to the diagnostic presumption and histopathologic confirmation of a rare subtype of classical Hodgkin lymphoma. Moreover, this case exemplifies a scenario where the classic “waxing and waning” clinical tendency is not pathognomonic of low grade lymphoproliferative disorders and can evidently be present in high grade lymphomas.

## Abbreviations

ANA, antinuclear antibodies; ANC, absolute neutrophil count; CT, computed tomography; CSF, cerebral spinal fluid; EBV, Epstein Barr virus; EBER, Epstein–Barr virus-encoded small RNAs; WBC, white blood cells; Hgb, hemoglobin; HL, Hodgkin lymphoma; LDCHL, lymphocyte depleted classical Hodgkin lymphoma; MRI, magnetic resonance imaging.
